# c-Myc regulates neural stem cell quiescence and activation by coordinating the cell cycle and mitochondrial remodeling

**DOI:** 10.1038/s41392-021-00664-7

**Published:** 2021-08-25

**Authors:** Chunhui Cai, Xinyu Hu, Peibin Dai, Tianran Zhang, Mei Jiang, Liefu Wang, Wanhao Hua, Yantao Fan, Xin-Xin Han, Zhengliang Gao

**Affiliations:** 1 Yangzhi Rehabilitation Hospital (Shanghai Sunshine Rehabilitation Center), Tongji University School of Medicine, Shanghai, China; 2 Institute of Geriatrics (Shanghai University), Affiliated Nantong Hospital of Shanghai University (The Sixth People’s Hospital of Nantong), School of Medicine, Shanghai University, Nantong, China; 3 Shanghai Engineering Research Center of Organ Repair, School of Medicine, Shanghai University, Shanghai, China; 4 Xinyang Vocational and Technical College, Xinyang, Henan China; 5 Shanghai Key Laboratory of Craniomaxillofacial Development and Diseases, Shanghai Stomatological Hospital, Fudan University, Shanghai, China

**Keywords:** Quiescence, Neural stem cells

**Dear Editor**,

In the adult brain, the transition from neural stem cell (NSC) quiescence to activation is a focal regulatory point for neural regeneration and dysregulations in the transition lead to brain disorders.^1,2^ During this transition, both cell cycle states and metabolism including mitochondria undergo extensive reprogramming. A deep understanding of such comprehensive changes is a prerequisite for a holistic picture of physiology and may aid disease combats. However, mechanisms coordinating their spatiotemporal regulations remains a least understood subject. A key challenge is to establish suitable experimental systems and hunt for master controllers coordinating cell cycle and metabolic reprogramming during the transition.

c-Myc, a multifunctional transcription factor with thousands of targets, plays a central role in cell cycle and metabolic coordination in cancer cells.^3^ c-Myc-mediated metabolic switching is also critical for stem cell fate decisions.^4^ RNA in situ data from Allen Brain Atlas suggest that *c-Myc* transcripts are expressed in the adult hippocampus, where adult neurogenesis actively takes place (Supplementary Fig. S1a and b). Further morphological and immunostaining analyses revealed that c-Myc was expressed in the stem cell and progenitor compartments (Fig. 1a and Supplementary Fig. S1d). Closer examination revealed that quiescent NSCs (qNSCs), positive for GFAP (cytoplasmic) and SOX2 but not for Ki67 (nuclear), were predominantly c-Myc^Low^ cells (71% ± 5%). Only a small subset was c-Myc^high^ cells (28% ± 1%) (Fig. 1b), presumably representing the subpopulation of quiescent NSCs primed for activation. In contrast, the majority of activated NSCs (aNSCs) and proliferative progenitors, positive for Sox2/Ki67 but not for GFAP, were c-Myc^high^ cells (67% ± 1%), and a smaller subset were c-Myc^Low^ cells (32% ± 1%; *n* = 3) (Fig. 1b). In support of the above, analysis of published scRNA-seq data confirmed a high expression of c-Myc in aNSCs but not in qNSCs (Supplementary Fig. S1c).

**Fig. 1:**
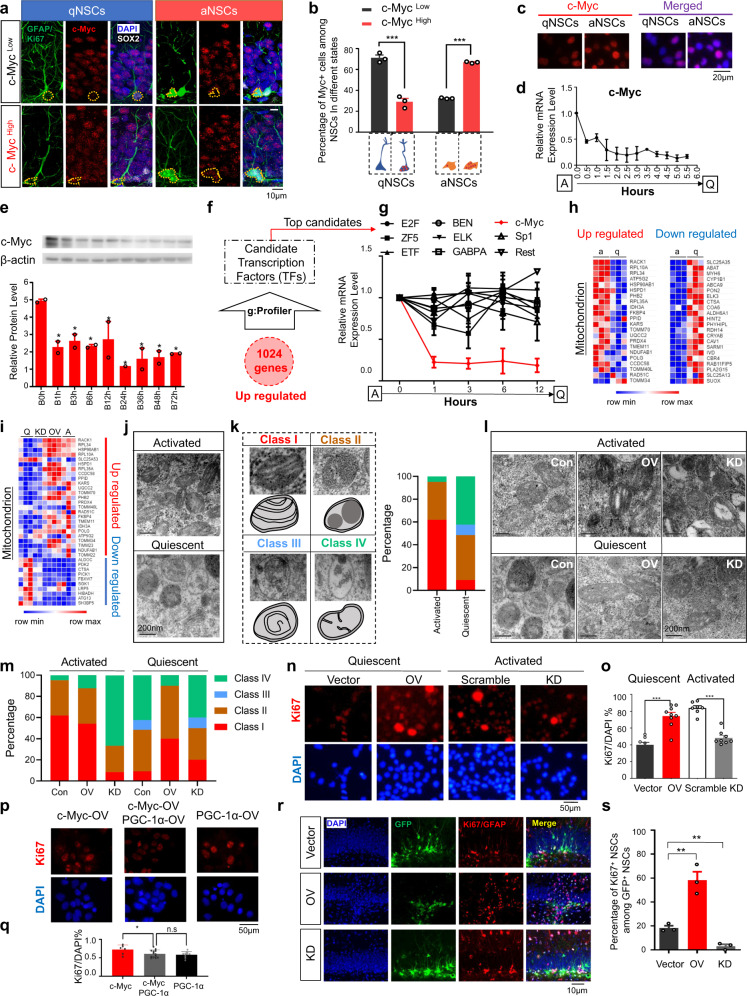
c-Myc regulates neural stem cell quiescence and activation by coordinating the cell cycle and mitochondrial remodeling. **a** Confirmation of c-Myc expression (red) in the NSC and progenitor compartments by co-staining with GFAP (green)/Sox2 (white)/Ki67 (green) and DAPI (blue). qNSCs: GFAP^+^/Sox2^+^/Ki67^−^ and aNSCs: GFAP^−^/Sox2^+^/Ki67^+^. **b** Differential expression of c-Myc in qNSCs and aNSCs. qNSCs were predominantly c-Myc ^low^ cells (71% ± 5%) (*n* = 3), and aNSCs were mostly c-Myc ^high^ cells (67% ± 1%) (*n* = 3). **c** c-Myc expression dynamics in NSCs in vitro. Left: c-Myc staining in qNSCs and aNSCs; Right: c-Myc staining merged with DAPI. **d** RT-qPCR analysis showed rapid repression of *c-Myc* transcription occurred immediately (6 h) upon quiescence induction (*n* = 3). Half an hour after quiescence induction, *c-Myc* transcripts already decreased by 2-fold and at 1.5 to 6 h, had decreased by 3- to 10-fold. **e** Western blot analysis showed decreasing c-Myc protein level during the transition to quiescence. **f** Schematic of the transcription factor binding motif enrichment analysis of the promoters from upregulated (1024) genes. **g** Expression dynamics of the top enriched transcription factors in NSCs upon quiescence induction, determined by RT-qPCR analysis (*n* = 3). For each gene, its expression value at the first timepoint was used as the normalizing control for its own series. **h** Expression patterns of mitochondrial genes in aNSCs v qNSCs. **i** Expression patterns of mitochondrial genes in aNSCs (A), c-Myc KD aNSCs (KD), qNSCs (Q), and c-Myc OV qNSCs (OV). **j** Morphological and size changes of mitochondria in aNSCs and qNSCs determined by TEM. **k** Classification of mitochondria by the status of cristae and distribution of different types of mitochondria in aNSCs and qNSCs. **l** c-Myc overexpression and knockdown altered mitochondrial morphology and size. **m** Distribution of different types of mitochondria with c-Myc overexpression and knockdown. **n** Ki67 (red) and DAPI (blue) staining in aNSCs and qNSCs transfected with vector (*n* = 8 fields), c-Myc OV (*n* = 9 fields), scramble (*n* = 7 fields), and c-Myc KD (*n* = 8 fields). **o** Increased proliferation with c-Myc-OV qNSCs (*n* = 9 fields) and decreased proliferation with c-Myc KD aNSCs (*n* = 8 fields) as determined by Ki67 staining. **p** Ki67 (red) and DAPI (blue) staining in NSCs with PGC-1α overexpression. **q** Ablation of the c-Myc-mediated increase in proliferation by the mitochondrial biogenesis regulator PGC-1α (*n* = 6, 10, and 13 fields). **r** Co-staining of GFP (green), Ki67 (red), Sox2 (white), and DAPI (blue). **s** Lentivirus-mediated c-Myc OV drastically increased but c-Myc KD drastically decreased hippocampal NSC proliferation in vivo (*n* = 3). All data are presented as the mean ± SEM values. An unpaired *t*-test was used to analyse the difference between the two groups. *P < 0.05; **P < 0.01; ***P < 0.002; n.s, no significance

To facilitate mechanistic investigation, we switched to an in vitro HCN (hippocampal NSC) culture system^5^ that could mimic the in vivo reversible NSC quiescence and activation transition (Supplementary Fig. S2). In vitro the expression of c-Myc was highly dynamic during the transition, much higher in aNSCs than in qNSCs (Fig. 1c and Supplementary Fig. S1e). Half an hour after quiescence induction, *c-Myc* transcripts already decreased by 2-fold and at 1.5–6 hours, by 3- to 10-fold (Fig. 1d and Supplementary Fig. S1f). A similar but less dramatic decrease was observed with c-Myc proteins (Fig. 1e).

Meanwhile, transcriptome analysis revealed that 1024 genes were upregulated and 1818 genes were downregulated in aNSCs compared with qNSCs (Supplementary Fig. S3a, b). Further transcription factor binding motif enrichment analysis of promoters of the upregulated genes identified a list of candidates (Fig. 1f). Among them, *c-Myc* was not only the most highly expressed but also the most dynamically expressed during NSC quiescence and activation transition (Fig. 1g and Supplementary Fig. S3c, d). Of the 1024/1818 genes differentially expressed, over 93% (956/1692) were found to have confirmed c-Myc binding in the ENCODE database (Supplementary Fig. S3e). Among them the cell cycle and mitochondrial pathway genes were highly enriched (Fig. 1h and Supplementary Fig. S3f–h). We randomly took 6, *Hsp90ab1, Hspd1, Tmem11* from the upregulated group and *Cyp1b1, Hint2, Rdh14* from the downregulated one and all had a modestly stronger binding of c-Myc to their promoters in expected NSC states (Supplementary Fig. S3i, j). Taken together, these results suggest that c-Myc may modulate NSC quiescence and activation through coordinating the cell cycle status and mitochondrial activity.

To confirm that, we took advantage of the in vitro culture model system to overexpress and knockdown c-Myc and performed transcriptome analysis (Supplementary Fig. S4a, b). Again, the cell cycle and mitochondrial pathway genes were among the most significantly altered by c-Myc (Supplementary Fig. S4c, d and Fig. 1i). Consistent with the molecular changes, examination via transmission electron microscopy (TEM) revealed that mitochondrial cristae were thick and well organized in aNSCs but were sparse, fragmented and disorganized in qNSCs (Fig. 1j, k and Supplementary Fig. S5a). Compared with those in aNSCs, mitochondria in qNSCs were swollen and immature, and as a result, their activity was also compromised (Supplementary Fig. S5b). Approximately 35% of mitochondria in qNSCs but only less than 10% in aNSCs were inactive (Supplementary Fig. S5b). ATP production in qNSCs was also significantly reduced (Supplementary Fig. S5c). Consistent with the molecular changes seen above, c-Myc overexpression in qNSCs reverted the mitochondrial morphological changes and the disappearance of cristae that typically occurs upon quiescence induction (Fig. 1l, m). In contrast, c-Myc knockdown in aNSCs shifted mitochondria towards a more quiescence-like state (Fig. 1l, m and Supplementary Fig. S5d). Consequentially c-Myc overexpression increased but shRNA knockdown decreased ATP production (Supplementary Fig. S5e).

In line with shifts in mitochondrial remodeling, c-Myc overexpression enhanced proliferation and reduced the percentage of cells in the G0 phase under the quiescent condition but not under the activated one (Fig. 1n, o and Supplementary Fig. S6a, b). In contrast, c-Myc knockdown caused cell cycle arrest and increased the percentage of cells in the G0 phase under the activated condition but not under the quiescent one (Fig. 1n, o and Supplementary Fig. S6a, b). Importantly, both PGC-1α, a regulator of mitochondrial biogenesis and NaN_3_, an inhibitor of mitochondrial activity, effectively abolished c-Myc-mediated NSC activation (Fig. 1p, q and Supplementary Fig. S6c–e), suggesting that c-Myc regulates quiescence and activation transition at least partially through controlling mitochondrial remodeling.

We then performed in vivo stereotaxic injection of Vector-, c-Myc OV- and KD-GFP lentiviruses into adult hippocampi. After confirming successful c-Myc overexpression and/or knockdown in vivo by co-staining c-Myc with GFP (Supplementary Fig. S6f), we found that c-Myc OV drastically increased proliferation and c-Myc KD had the opposite effect (Fig. 1r). The percentage of Ki67^+^ cells among GFP^+^ ones was much higher in the c-Myc OV group (58% ± 10%; *n* = 3) and lower in the c-Myc KD group (3% ± 2%; *n* = 3) than in the control group (18% ± 3%; *n* = 3) (Fig. 1s). Their morphologies were drastically different too (Supplementary Fig. S6g). Essentially all c-Myc OV-GFP cells have lost RGL morphology and adopted aNSC/early progenitor morphology while the Vector-GFP ones manifested both. The c-Myc KD-GFP cells mainly kept RGL morphology (Supplementary Fig. S6g, h). This is indeed expected and shows that 3-day c-Myc overexpression was enough to activate the infected qNSCs which could not stay quiescent with high expression of c-Myc and 3-day c-Myc knockdown was sufficient to arrest the infected cells which rarely become activated without c-Myc upregulation.

In summary, the present study reveals a central role of c-Myc in NSC quiescence and activation homeostasis through coordinating metabolic reprogramming (e.g., mitochondrial remodeling) with the cell cycle status. Our work also exemplifies how to utilize in vitro modeling to obtain an integrated understanding of how quiescence and activation are coordinated through master cell cycle and metabolism controllers such as c-Myc. It is now of high interest to utilize the in vitro culture model system to delineate in a stage-wise manner and on a genome-wide scale how different master controllers (e.g., c-Myc, REST, E2F1, and Ascl1) antagonize and coordinate to balance NSC quiescence and activation in time and space. A systematic understanding of the transition and its dynamics shall help reveal novel strategies for neural regeneration therapies.

## Supplementary information

Supplementary Materials for c-Myc regulates neural stem cell quiescence and activation by coordinating the cell cycle and mitochondrial remodelling

## Data Availability

The datasets used and/or analyzed to support the findings of this study are available in this paper or the Supplementary information. Any other raw data that support the findings of this study are available from the corresponding author upon reasonable request.

## References

[1] Urban N, Blomfield IM, Guillemot F (2019). Quiescence of adult mammalian neural stem cells: a highly regulated rest. Neuron.

[2] Bond AM, Ming GL, Song H (2015). Adult mammalian neural stem cells and neurogenesis: five decades later. Cell Stem Cell.

[3] Hsieh AL, Walton ZE, Altman BJ, Stine ZE, Dang CV (2015). MYC and metabolism on the path to cancer. Semin. Cell Dev. Biol..

[4] Cliff TS (2017). MYC controls human pluripotent stem cell fate decisions through regulation of metabolic flux. Cell Stem Cell.

[5] Mira H (2010). Signaling through BMPR-IA regulates quiescence and long-term activity of neural stem cells in the adult hippocampus. Cell Stem Cell.

