# Supplementary material for: c-Myc regulates neural stem cell quiescence and activation by coordinating the cell cycle and mitochondrial remodeling
Source: Signal Transduct Target Ther. 2021 Aug 25;6:306. doi: 10.1038/s41392-021-00664-7 (PMC8385022; doi:10.1038/s41392-021-00664-7)
Supplement: Supplementary file 1 — Supplementary Materials for c-Myc regulates neural stem cell quiescence and activation by coordinating the cell cycle and mitochondrial remodelling [file 41392_2021_664_MOESM1_ESM.docx]

Supplementary Materials for

c-Myc regulates neural stem cell quiescence and activation by coordinating the cell cycle and mitochondrial remodelling

Chunhui Cai ^1, 2, 3^, Xinyu Hu ^1, 2, 3^, Peibin Dai ^1^, Tianran Zhang^1^, Mei Jiang^1^, Liefu Wang^4^, Wanhao Hua^1, 2, 3^, Yantao Fan ^1, 2, 3^, Xin-Xin Han ^5^, Zhengliang Gao ^1, 2, 4^

1. *Yangzhi Rehabilitation Hospital (Shanghai Sunshine Rehabilitation Center), Tongji Univeirsity School of Medicine, 201619 Shanghai, P. R. China*
2. *Institute of Geriatrics (Shanghai University), Affiliated Nantong Hospital of Shanghai University (The Sixth People’s Hospital of Nantong), School of Medicine, Shanghai University, 226011 Nantong, P. R. China*
3. *Shanghai Engineering Research Center of Organ Repair, School of Medicine, Shanghai University, 200444 Shanghai, P. R. China*
4. *Xinyang Vocational and Technical College, 464000 Xinyang, Henan, P. R. China*
5. *Shanghai Key Laboratory of Craniomaxillofacial Development and Diseases, Shanghai Stomatological Hospital, Fudan University, 200001 Shanghai, P. R. China*

These authors contributed equally: Chunhui Cai, Xinyu Hu, Peibin Dai

**Corresponding Authors**

* E-mail: Xin-Xin Han [(xxhan@fudan.edu.cn)](mailto:(xxhan@fudan.edu.cn))

* E-mail: Zhengliang Gao [(zhengliang_gao@tongji.edu.cn)](mailto:(zhengliang_gao@tongji.edu.cn))

**This PDF file includes:**

Materials and Methods

Supplemental Figures S1 to S6

**Material and Methods**

**Animals**

Adult male SD rats (6-7 weeks old) were purchased from Shanghai SLAC Laboratory Animal Co., Ltd., and housed in the experimental animal center of Tongji University. All animal care and experimental procedures were reviewed and approved by the Animal Committee of the school.

**Immunofluorescence staining of tissues**

Immunofluorescence staining was performed on mounted sections as previously described.^1^ All mounted sections were subjected to antigen retrieval with 0.01 M citric acid (pH 6.0) at 95 °C for 15 mins. After 30 min of permeabilization in 0.25% PBST, brain sections were blocked with 5% normal donkey serum at RT for 1 hour and were then incubated with primary antibodies at 4°C overnight. Secondary antibody incubation was performed at RT for 2 hr. The primary antibodies were mouse anti-Ki67 (1:500, BD Biosciences, 550609), goat anti-Sox2 (1:500, Santa Cruz, sc-17320), mouse anti-GFAP (1:750, Millipore, MAB360) and rabbit anti-c-Myc (1:1000, Abcam, AB39688). All secondary antibodies were purchased from Jackson ImmunoResearch Laboratories and used at a 1:1000 dilution. DAPI was used to counterstain nuclei. Images were acquired, and cell counting was performed with a Nikon confocal microscope (Nikon A1R).

**Cell culture and Sample collection**

The Hippocampus neural stem cells (HCN)-A94 cells were a gift from Prof. Fred H Gage (Salk Institute, US) and were cultured as previously described.^2^ Briefly, cells were maintained in DMEM/F12 medium supplemented with N2 and 20 ng/mL bFGF (growth medium) for activation and proliferation or 20 ng/mL bFGF and 50 ng/mL BMP4 (quiescence medium) for quiescence induction and maintenance. Neuronal, astrocytic and oligodendrocytic differentiation were respectively induced with 1 µM retinoic acid and 5 µM forskolin, 50 ng/mL BMP4 and 10^5^ units of LIF, and 500 ng/mL IGF-I.^3^

For RT-qPCR analysis, HCN cells were seeded in 6-well plates at 0.5×10^6^ cells/well in growth medium and overnight were switched to quiescence medium. At 0, 0.5, 1, 2, 3, 6, 12, 24, 48 and 72 hrs upon quiescence induction, cells were then scrapped off the wells, collected and extracted in RNA-TRIzol. RT-qPCR analysis was carried out with RNA samples at indicated time points. For RNA-seq analysis, HCN cells were seeded in 10 cm tissue culture dishes at 3×10^6^ cells/dish in growth medium and overnight were switched to growth medium or quiescence medium. Three days after, cells were collected with RNA extracted with RNA-Trizol. For Western blot analysis, HCN cells in 10 cm tissue culture dishes were washed with PBS and then scrapped off the dishes and collected as cell pellets and stored at -80°C. For electron microscopy, HCN cells was cultured in growth and/or quiescence mediums for 72 hrs. For cell proliferation assay by Ki67 staining and cell cycle analysis by flow cytometry, the same number of the cells for each experimental group were seeded in 24-well and/or 6-well plates in the indicated medium and at 48hrs were collected for experimentation.

**Immunofluorescence staining of cells**

Cells were fixed with 4% (w/v) paraformaldehyde for 15 min and permeabilized with 0.25% Triton- X-100 in 1× PBS for 15 min at room temperature (RT). After 1 hr of blocking with 3% BSA at RT, primary antibody incubation was performed at 4°C overnight with secondary antibody staining at RT for 2 hr. The primary antibodies were rabbit anti-Ki67 (1:1000, Thermo Fisher, MA5-14520), goat anti-Sox2 (1:1000, Santa cruz, sc-17320), mouse anti-Nestin (1:1000, Millipore, MAB353), rabbit anti-c-Myc (1:1000, Abcam, AB39688), mouse anti-RIP (1:500, Developmental Studies Hybridoma Bank), rabbit anti-Tuj1 (1:750, Covance, PRB-435P), and rabbit anti-GFAP (1:1000, Dako, N1506). Secondary antibodies were purchased from Jackson ImmunoResearch Laboratories and used at a 1:1000 dilution. DAPI was used for counterstaining. Images were acquired with a Nikon ECLIPSE Ti microscope. Cell counting was done with ImageJ cell counter plugins. The ratio of Ki67^+^/DAPI-labelled cells represents the proliferation rate. All experiments were repeated independently at least three times.

**RNA isolation and quantitative real-time PCR (RT-qPCR)**

RNA was extracted using TRIzol reagent. The RNA integrity and concentration were assessed with a Qubit®3.0 Fluorometer (Life Technologies, CA, USA) and a RNA Nano 6000 Assay Kit in a Bioanalyzer 2100 system (Agilent Technologies, CA, USA). A total amount of 2 μg RNA per sample was converted to cDNA using a FastQuant RT Kit (TIANGEN, KR106). Quantitative PCR was carried out in a BioRad T100 PCR system using SuperRealPreMix Plus (TIANGEN, FP205) and appropriate primers. Expression levels of target genes were quantified with respect to 18S and GAPDH as the internal controls using the comparative CT method.

**RNA sequencing**

A total amount of 2 μg RNA per sample was used, and sequencing libraries were prepared and indexed using an NEBNext®Ultra™ RNA Library Prep Kit for Illumina® (#E7530L, NEB, USA) following the manufacturer’s recommendations. Library concentrations were determined with a Qubit® RNA Assay Kit in Qubit® 3.0, and the libraries were then diluted to 1 ng/µL. Insert sizes were assessed using the Agilent Bioanalyzer 2100 system (Agilent Technologies, CA, USA), and insert concentrations were quantified using a StepOnePlus™ Real-Time PCR System (valid concentration＞10 nM). Clustering of the index-coded samples was performed in a cBot cluster generation system using an Illumina HiSeq PE Cluster Kit v4-cBot-HS. Sequencing was performed on the Illumina HiSeq 4000 platform with 150-bp paired-end reads.

**c-Myc Chip-PCR**

Quiescent and activated HCN cells were collected after 48hrs incubation in growth medium or quiescence medium. NovoNGS CUT&TAG 3.0 kit (Cat. NO: N259-YH01, Novoprotein Scientific Inc.) was used together with c-Myc primary antibody (9E10: sc-40, Santa Cruz Biotechnology) to collect the c-Myc binding DNA fragments. Q-PCR was performed afterward with *Hsp90ab1(*5’: CTCGGCTTTCTCGTCAAGGT; 3’: ATGAGATGTTGGCGGAGACC*), Hspd1(*5’: ACTTGTCCAACGGTGCTTCA; 3’: GGTTTCGTGTGCTTTGTGCT*), Tmem11 (*5’: AAAGAGTCTCCGAGTCGCAA; 3’: GTGGTAGGCGCTTTCTTTCC*), Cyp1b1 (*5’: GCTGTGGACTGTCTGCACTA; 3’: TTGCACACCTGAGTGTCACG*), Hint2 (*5’: AACCAGGCTGTAACAGAGGG; 3’: ACCCCACCGTTTCTCTTCAG*)* and *Rdh14 (*5’: GTCATCATGGGATGTCGGGA; 3’: AGACCCACACCTGTAGCAGT*) primers.*

**Gene overexpression and knockdown**

The c-Myc-over expressing lentiviral construct was a gift from Dr. Lei Xiao (Zhejiang University, China). To generate the c-Myc shRNA knockdown construct (c-Myc-KD), the shRNA sequence CCTGAAACAGATCAGCAACAA was inserted into the pLVX-shRNA vector containing the ZsGreen expression cassette (shRNA2). A scrambled sequence (TTCTCCGAACGTGTCACGT) was used as the negative control (shRNA scramble). c-Myc OV, KD and control lentiviruses were produced in 293T cells and used to infect NSCs. Real-time PCR and Western blot analyses were performed to confirm successful c-Myc overexpression and knockdown. The PGC1-α overexpression construct (PGC1-α-OV) was a gift from Dr. Zhengji Gan (Nanjing University, China).

**Western blotting**

Total cell lysates were prepared in RIPA buffer containing 0.1% SDS, protease inhibitor cocktails and DNases. Proteins were separated by SDS–PAGE and transferred onto polyvinylidene difluoride membranes. Blocking and primary antibody incubation were performed in 3% BSA. Beta-actin was used as the loading control. The primary antibodies were rabbit anti-c-Myc (1:1000, Santa Cruz, sc-764) and mouse anti-beta-Actin (1:3000, Sigma, A2228).

**Electron microscopy**

Samples were prepared as previously described.^4^ Briefly, cells were fixed with 2% glutaraldehyde in PBS at 4°C overnight. After washing five times in PBS for 5 min each, cells were post-fixed with 1% osmium tetroxide (OsO_4_) for 1 hr at RT. For dehydration, a graded series of 30%, 50%, 70% and 90% acetone was used with immersion for 30 min per step, and the final dehydration was performed with immersion in 100% acetone three times for 10 min each at RT. Cells were then infiltrated in 3:7 (v/v) acetone:epon for 2 hr and then in 7:3 acetone:epon at RT overnight. Samples were then embedded with 100% epon for 2 hr and polymerized with 100% epon at 60 °C for 48 hr. Sections of 70 nm were supported on copper grids and post-stained with uranyl acetate and lead citrate. Images were acquired using a transmission electron microscope (JEM-1230).

**Measurement of mitochondrial membrane potential and activity**

MitoTracker Green FM dye enters, accumulates in, and stains mitochondria. MitoTracker Deep Red FM emits fluorescence only when it enters actively respiring mitochondria and becomes oxidized. Thus, the ratio of red FM/green FM indicates mitochondrial activity. MitoTracker Green FM and MitoTracker Deep Red FM double staining was performed to quantify the total and functional mitochondria, respectively. After 30 min of staining with 100 nM MitoTracker Green FM and 100 nM MitoTracker Deep Red FM, cell samples were analysed by flow cytometry (BD).

**Cellular ATP measurement**

The CellTiter-Glo luminescent cell viability assay determines cell viability based on the amount of ATP presence, an indicator of metabolically active cells. Thus, we utilized this assay to measure cellular ATP production following the manufacturer’s instructions. CellTiter-Glo luminescent signal was detected by the GloMax Multiluminometer with an integration time of 0.5 second.

**Flow cytometric cell cycle analysis with Hoechst and Pyronin Y staining**

Cells were trypsinized and collected. After 5 min of centrifugation at 500g, cell pellets were gently resuspended in 100 µL of PBS and washed 3 times with PBS. Cold (-20°C) 75% ethanol was slowly added dropwise to the cell suspension for fixation. Cell staining was performed with 0.5 μg/mL Hoechst 33258 in 300 µL of PBS at RT for 15 min and 0.5 μg/mL Pyronin Y on ice for 20 mins.

**Stereotactic injection of virus**

After one week of acclimation, rats were anesthetized with 2.5% pentobarbital (1 mL/500 g) and placed in a stereotaxic apparatus. Vector, c-Myc OV and KD-GFP lentiviruses were injected into the hippocampal dentate gyrus (AP, -4 mm; MDL, -1.9 mm; DV, +4 mm) of both cerebral hemispheres. The incisions were closed with the rats returned to the cage for recovery. Animals were sacrificed and perfused with 4% paraformaldehyde 3 days after stereotactic surgery. Rat brains were collected and post-fixed with 4% paraformaldehyde overnight and were then subjected to 20% and 30% sucrose gradient dehydration for 48 hr.

**Bioinformatic analysis**

Paired-end 150 bp reads were subjected to adapter trimming and removal of low-quality (Q<30) bases using Trimmomatic.^5^ The trimmed reads were mapped to the rat genome (rn6) using HISAT2.^6^ Quantification was carried out with StringTie^7^ with normalization to fragments per kilobase per million mapped reads (FPKM) values. Genes with FPKM values of less than 1 in all samples were removed. Differentially expressed genes (DEGs) were then identified with the ‘DEseq2’ package^8^ using a fold change of >2 and a *p*-value of <0.05 as the threshold criteria.

DEG enrichment analyses (Gene Ontology term and KEGG pathway) were performed with DAVID 6.8.^9^ Significant terms were plotted with the ‘ggplot2’ package in R (v3.5.3). Cluster analysis and heatmap generation were performed with the online analysis software Morpheus. Potential transcription factors (TFs) in a given gene list were analysed and identified with the web-based toolset g:Profiler.^10^ Related TF lists and position frequency matrices (PFMs) were downloaded from TRANSFAC^11^ and plotted with the ‘ggseqlogo’ package^12^ in R (v3.5.3). Venn diagrams were generated using an online Venn diagram tool.

A list of c-Myc target genes was downloaded from an ENCODE 3 Regulation track in the UCSC Genome Browser called Transcription Factor ChIP-seq Clusters (340 factors, 129 cell types) (data version: Nov 2018). The track contains binding sites of 338 transcription factors (340 in hg38) across 130 cell types (129 in hg38). According to this database, c-Myc binds to 16010 (peak score>500) total genes across the cell types.

**Significance analysis**

Statistical analyses were performed using Prism software (GraphPad) with two-tailed Student’s t-tests for comparisons between two groups and with analysis of variance (ANOVA) tests for comparisons among more than two groups. All data were presented as the mean ± standard deviation values where appropriate. A difference was considered to be significant if the p values was less than 0.05. Details on the sample numbers and significance levels were given in the figure legends.

**References**

1. Gao, Z. *et al*. Neurod1 is essential for the survival and maturation of adult-born neurons. *Nat Neurosci* **12**, 1090-10922 (2009).

2. Shen, B. *et al*. Genetically encoding unnatural amino acids in neural stem cells and optically reporting voltage-sensitive domain changes in differentiated neurons. *Stem Cells* **29**, 1231-1240 (2011).

3. Hsieh, J. *et al*. IGF-I instructs multipotent adult neural progenitor cells to become oligodendrocytes. *J Cell Biol* **164**, 111-122 (2004).

4. Fang, Y. *et al*. Smad5 acts as an intracellular pH messenger and maintains bioenergetic homeostasis. *Cell Res* **27**, 1083-1099 (2017).

5. Bolger, A. M., Lohse, M. & Usadel, B. Trimmomatic: a flexible trimmer for Illumina sequence data. *Bioinformatics* **30**, 2114-2120 (2014).

6. Kim, D., Langmead, B. & Salzberg, S. L. HISAT: a fast spliced aligner with low memory requirements. *Nat Methods* **12**, 357-360 (2015).

7. Pertea, M. *et al*. StringTie enables improved reconstruction of a transcriptome from RNA-seq reads. *Nat biotechnol* **33**, 290-295 (2015).

8. Love, M. I., Huber, W. & Anders, S. Moderated estimation of fold change and dispersion for RNA-seq data with DESeq2. *Genome Biol* **15**, 550 (2014).

9. Huang, D. W., Sherman, B. T. & Lempicki, R. A. Systematic and integrative analysis of large gene lists using DAVID bioinformatics resources. *Nat Protoc* **4**, 44-57 (2009).

10. Reimand, J., Kull, M., Peterson, H., Hansen, J. & Vilo, J. g:Profiler--a web-based toolset for functional profiling of gene lists from large-scale experiments. *Nucleic acids Res* **35**, 193-200 (2007).

11. Wingender, E. *et al*. TRANSFAC: an integrated system for gene expression regulation. *Nucleic acids Res* **28**, 316-319 (2000).

12. Wagih, O. ggseqlogo: a versatile R package for drawing sequence logos. *Bioinformatics* **33**, 3645-3647 (2017).

13. Kuan, L. *et al*. Neuroinformatics of the allen mouse brain connectivity atlas. *Methods* **73**, 4-17 (2015).

14. Bloss, E. B. *et al*. Single excitatory axons form clustered synapses onto CA1 pyramidal cell dendrites. *Nat Neurosci* **21**, 353-363 (2018).

**Supplementary Figures**

**
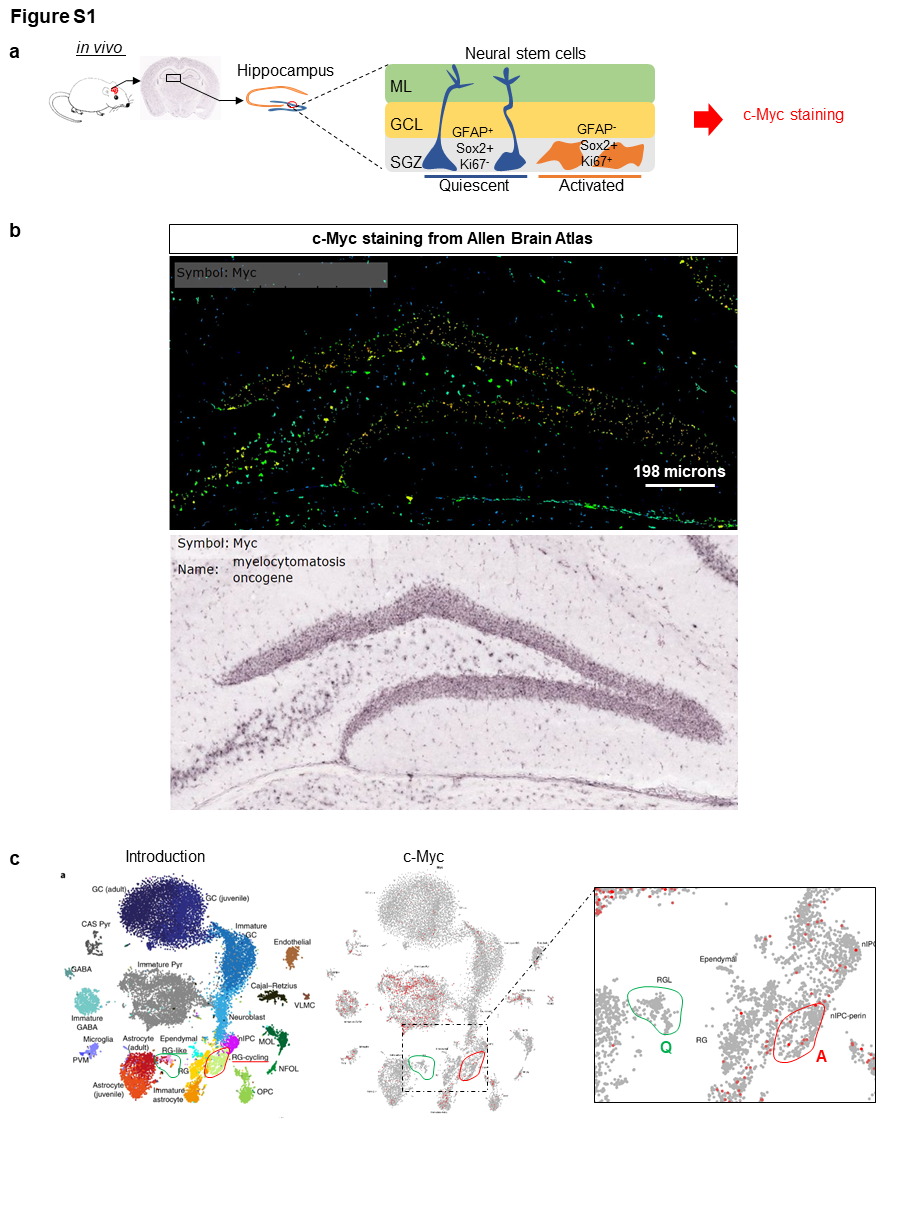

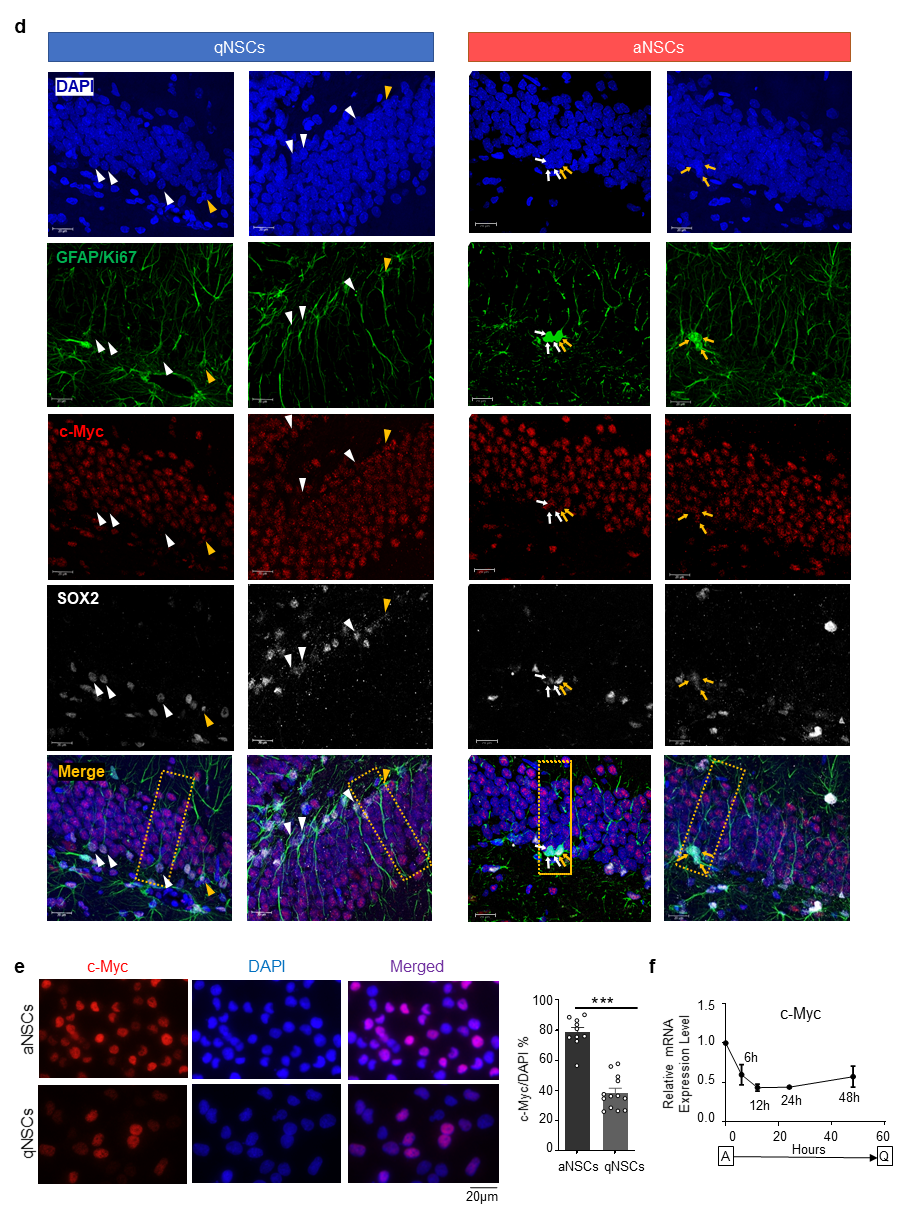
**

**Fig. S1 Dynamic expression of c-Myc during NSC quiescence and activation *in vitro* and *in vivo*.** **a** Schematic of the staining procedure for c-Myc expression level *in vivo*. **b** The expression pattern of c-Myc in hippocampus determined by RNA *in situ* from Allen Brain Atlas database.^13^ **c** single cell RNA sequencing analysis showing c-Myc was mainly expressed in aNSCs.^14^ Left panel: nomenclatures for cell subclusters, Green circle: RGL cells (qNSCs), Red circle: cycling-RG cells (aNSCs). Middle and right panels: c-Myc expression dynamics in subclusters and in RGL and cycling-RG cells. **d** c-Myc expression pattern in aNSC and qNSCs determined by co-staining of c-Myc (red) with GFAP (green)/Sox2 (white) /Ki67 (green) and DAPI (blue). White arrows and arrowheads stand for Myc^low^ cells while yellow arrows and arrowheads stand for Myc^high^ cells. **e** c-Myc expression dynamics in NSCs *in vitro*. The percentages of c-Myc positive cells/DAPI with aNSCs (n=10 fields) and qNSCs (n=13 fields) are presented as a bar chart. **f** Decreasing *c-*Myc transcript level during 48-hr of quiescence induction (n=3). All data are presented as the mean ± SEM values. An unpaired t-test was used to analyze the difference between the two groups. ****P* < 0.001.

**
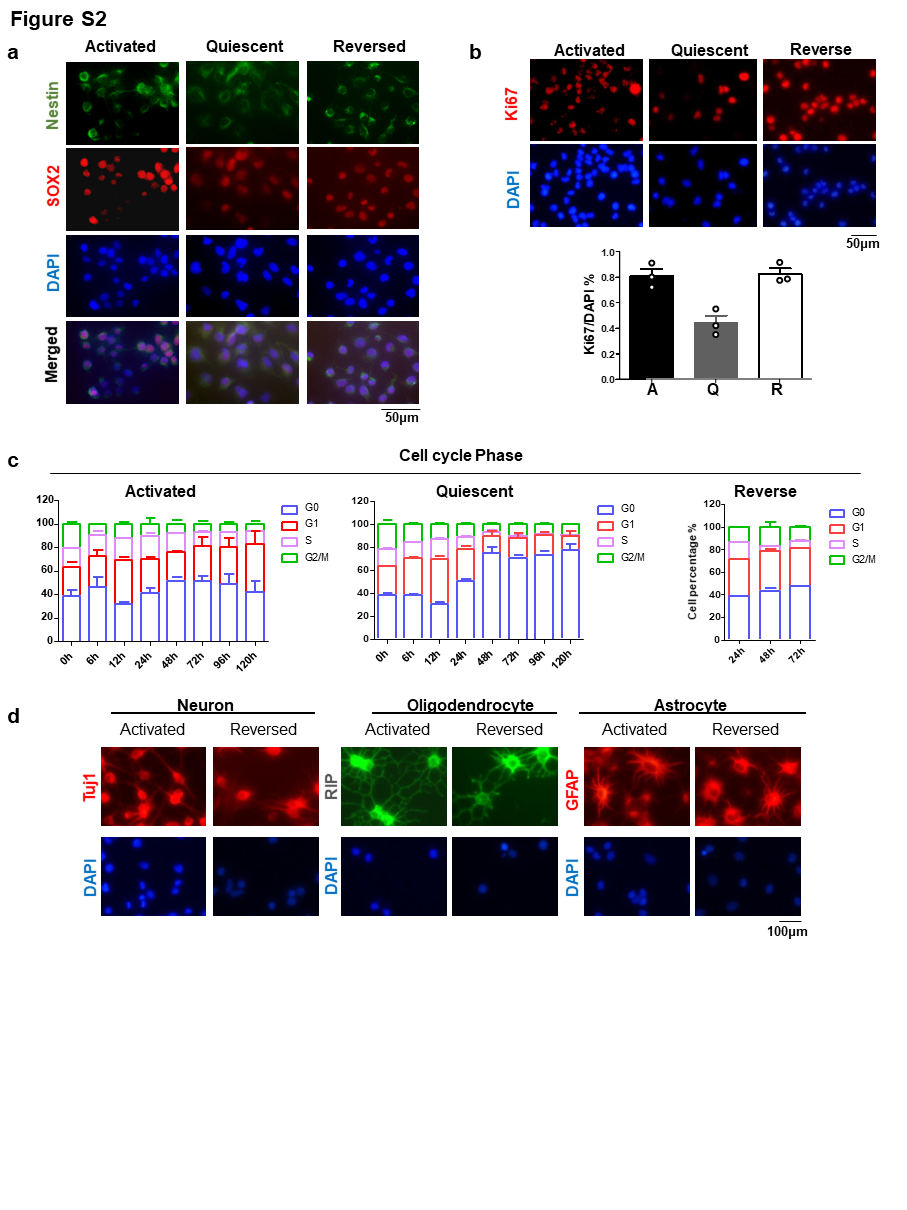
**

**Fig. S2 Successful establishment of a reversible NSC quiescence and activation culture model. a** Nestin and Sox2 staining in aNSCs, qNSCs and reversibly activated NSCs (rNSCs). **b** Determination and quantification of proliferation rates in aNSCs, qNSCs and reversibly activated NSCs (rNSCs) by Ki67 immunostaining (n=3). **c** Progressive induction of quiescence in aNSCs and reversible activation of qNSCs over time, as determined by PY-Hoechst flow cytometric analysis (n=3). **d** Differentiation potential of aNSCs and rNSCs, as confirmed by Tuji1, RIP and GFAP staining. All data are presented as the mean ± SEM values. Scale bars: 50 µm (a,b), 100 µm (d).

**
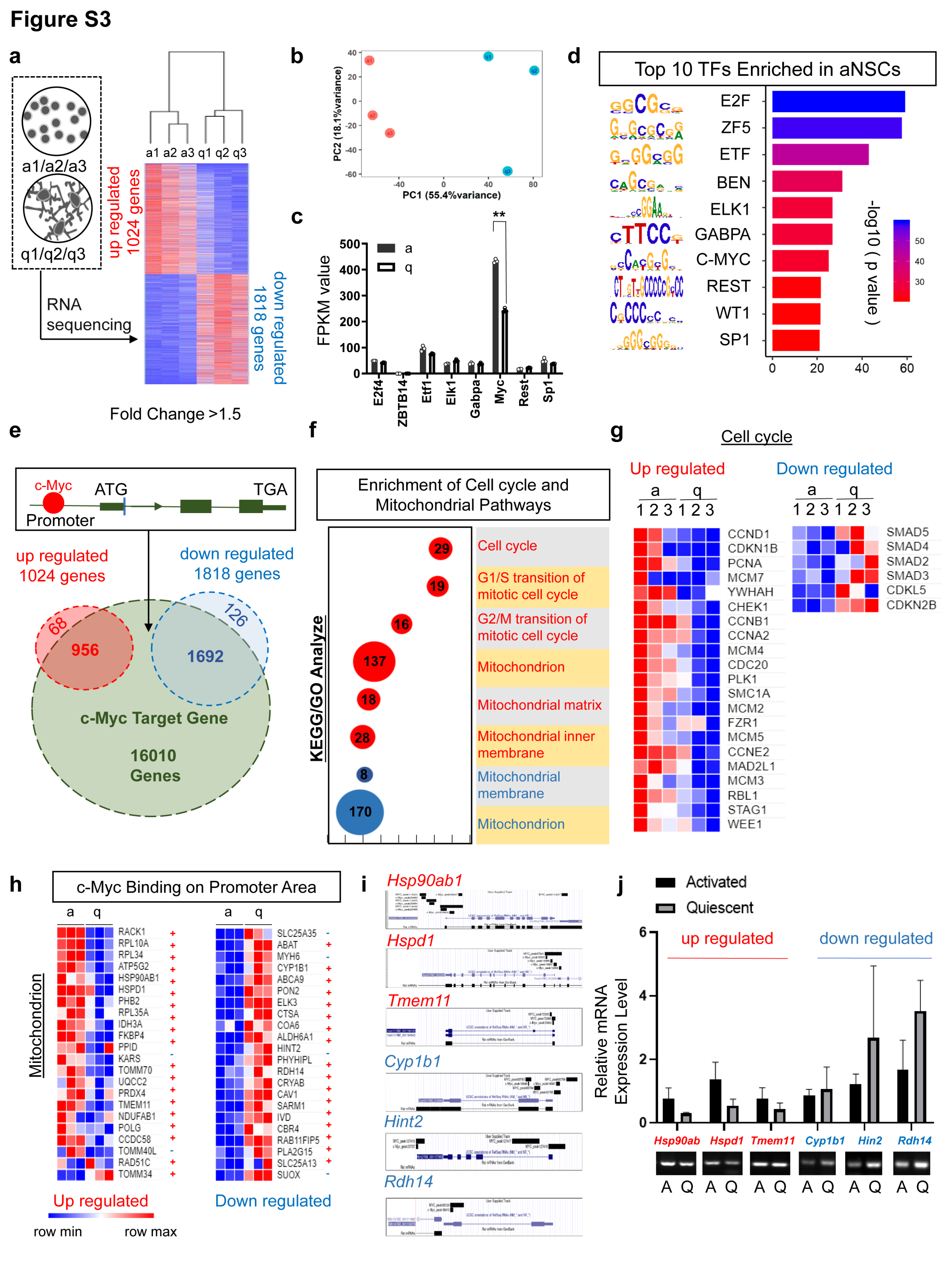
**

**Fig. S3 c-Myc identified as a master controller of NSC quiescence and activation. a** Hierarchical clustering of aNSC (a1/a2/a3) and qNSC (q1/q2/q3) transcriptomes identifies significantly up regulated (1024) and down regulated (1818) genes. **b** Principal component analysis (PCA) of transcriptomes of aNSCs (a1/a2/a3), qNSCs q1/q2/q3). **c** FPKM values of the relevant transcription factors from RNA sequencing data (n=3). **d** The top ten transcription factors identified by binding motif enrichment analysis of the promoters of the up regulated genes. **e** Schematic of the definition of c-Myc target genes. Venn diagram showing the numbers of overlapping up regulated (956) and down regulated (1692) genes with confirmed c-Myc target genes (16010) in the ENCODE database. **f** cell cycle and mitochondrial pathway genes were enriched in c-Myc target genes. Red circles: up regulated pathways, blue circles: down regulated pathways. Numbers in the circle: the numbers of genes. **g** Expression patterns of cell cycle genes in aNSCs v qNSCs. **h** Mitochondrial pathway genes with/out confirmed c-Myc binding to their promoters in ENCODE Project. **i** c-Myc binding at the genomic loci of representative genes including *Hsp90ab1, Hspd1, Tmem11, Cyp1b1, Hint2* and *Rdh14* gene loci*.*  **j** c-Myc Chip-qPCR results of representative genes *Hsp90ab1, Hspd1, Tmem11, Cyp1b1, Hint2* and *Rdh14*. All data are presented as the mean ± SEM values. An unpaired t-test was used to analyse the difference between the two groups. ***P* < 0.01.

**
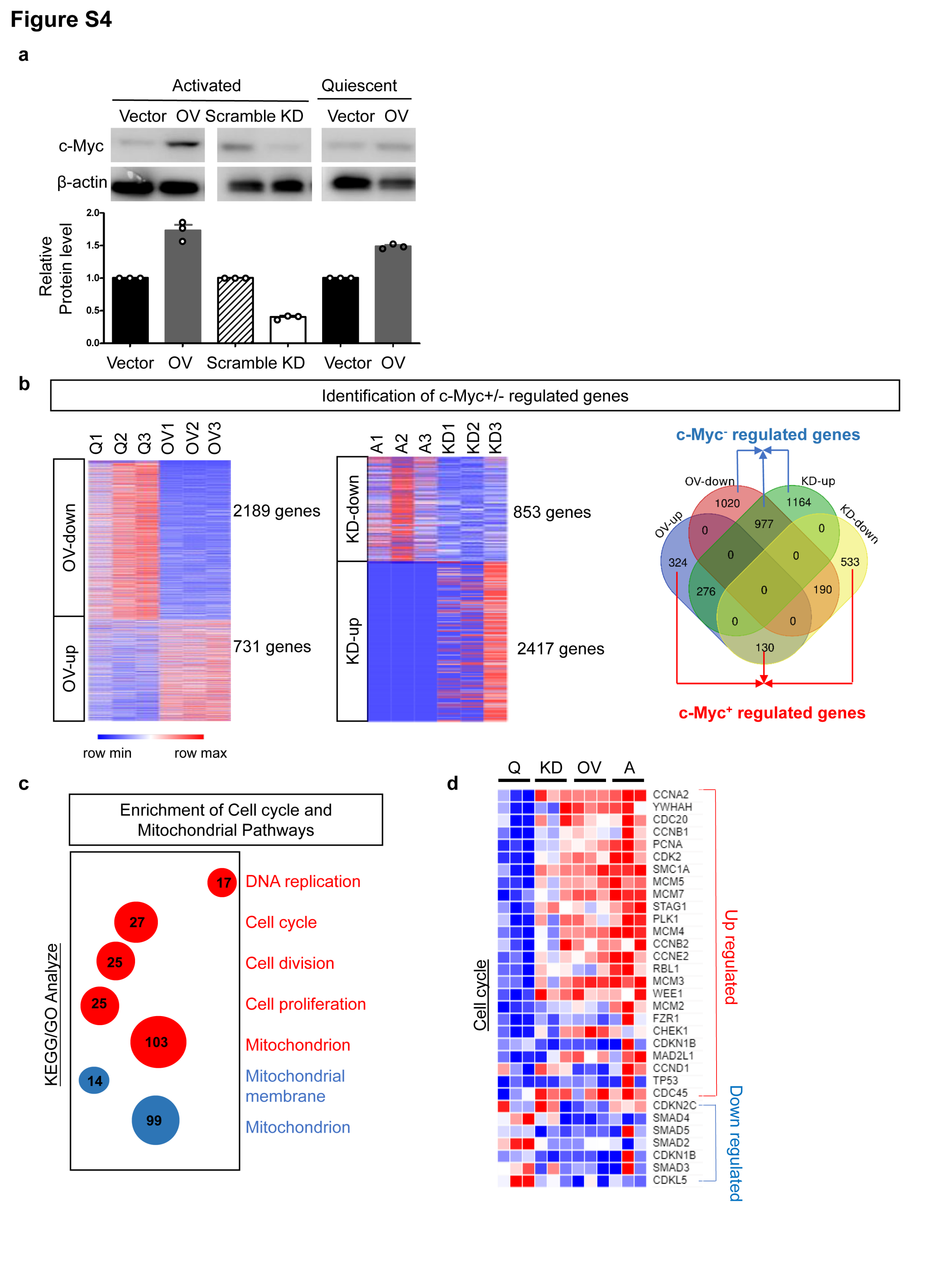
**

**Fig. S4 Coordination of cell cycle and mitochondrial metabolic genes by c-Myc. a** Successful overexpression (OV) and knockdown (KD) of c-Myc was confirmed by Western blot analysis (n=3). **b** Identification of c-Myc positive and negative (^+^/^-^) regulated genes. OV-down (2189) and OV-up (731) gene lists were obtained from a comparison between qNSCs (Q1/Q2/Q3) and OV qNSCs (OV1/OV2/OV3). KD-down (853) and KD-up (2417) gene lists were obtained from a comparison between aNSCs (A1/A2/A3) and KD aNSCs (KD1/KD2/KD3). The Venn diagram shows the overlap of these 4 gene lists. **c** KEGG and Gene Ontology (GO) analysis of c-Myc positive and negative (^+^/^-^) regulated genes. Red circles: upregulated pathways, blue circles: downregulated pathways. Numbers in the circles: the numbers of genes. **d** Expression patterns of cell cycle genes in OV qNSCs v qNSCs and KD aNSCs v aNSCs.


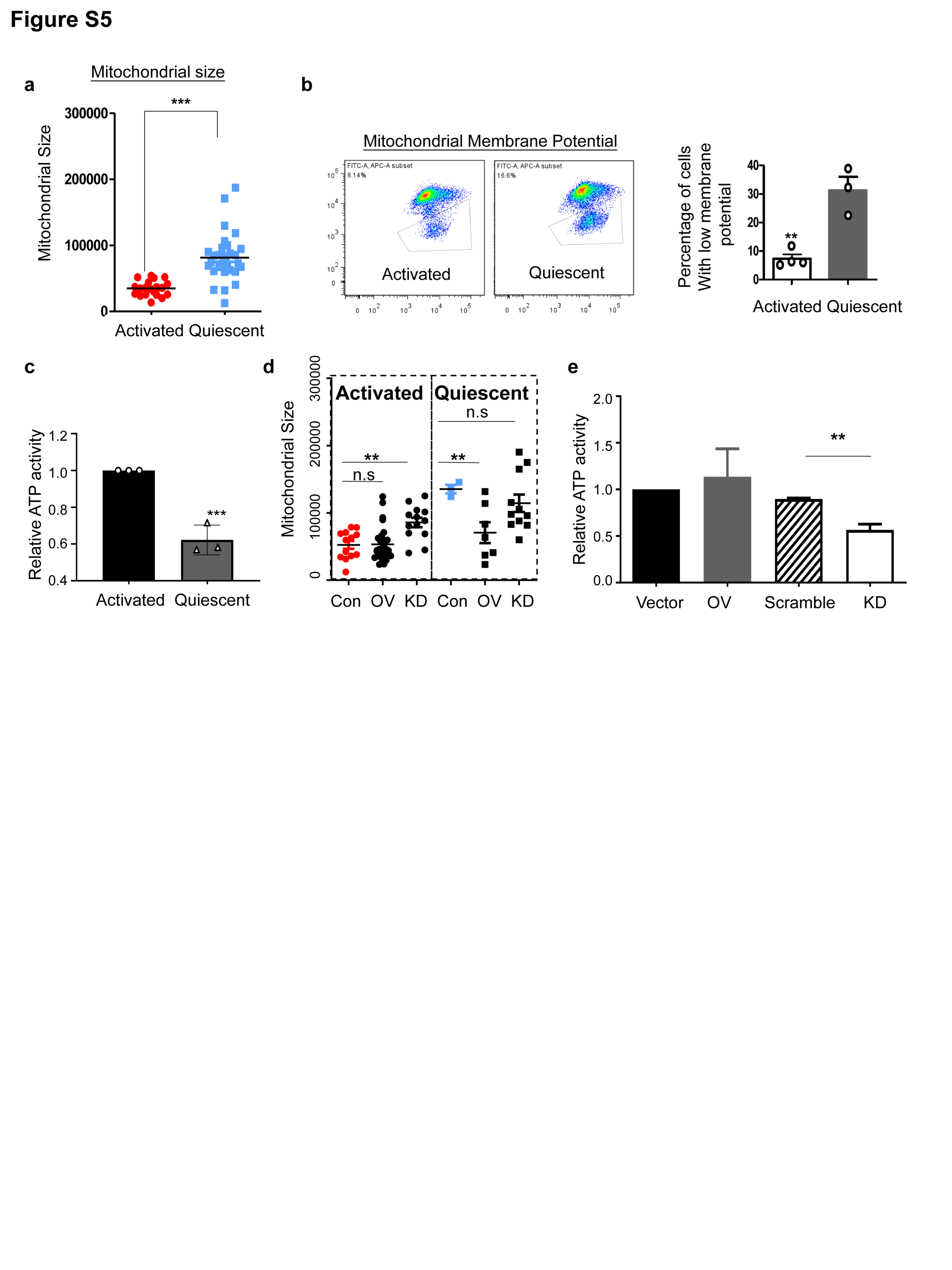


**Fig. S5 Mitochondrial remodelling during NSC quiescence and activation. a** The mitochondrial size was quantified in activated (n=20) and quiescent NSCs (n=31). The scatter plot shows that the average mitochondrial size was significantly increased in quiescent NSCs. **b** Decreased mitochondrial activity in qNSCs (n=3) compared to aNSCs (n=4), as determined by flow cytometric analysis of the mitochondrial membrane potential with MitoTracker Deep Red (mitochondrial membrane potential) and MitoTracker Green (total number of mitochondria). **c** ATP production in aNSCs and qNSCs. **d** Scatter plots showing the mitochondrial sizes in OV/KD activated (n=13, 29 and 13) and OV/KD quiescent NSCs (n=3, 7, and 11). **e** ATP production increased by c-Myc overexpression and decreased by its shRNA knockdown (n=3). All data are presented as the mean ± SEM values. An unpaired t-test was used to analyse the difference between the two groups. ***P*<0.01; ****P*<0.002; n.s, no significance.

**
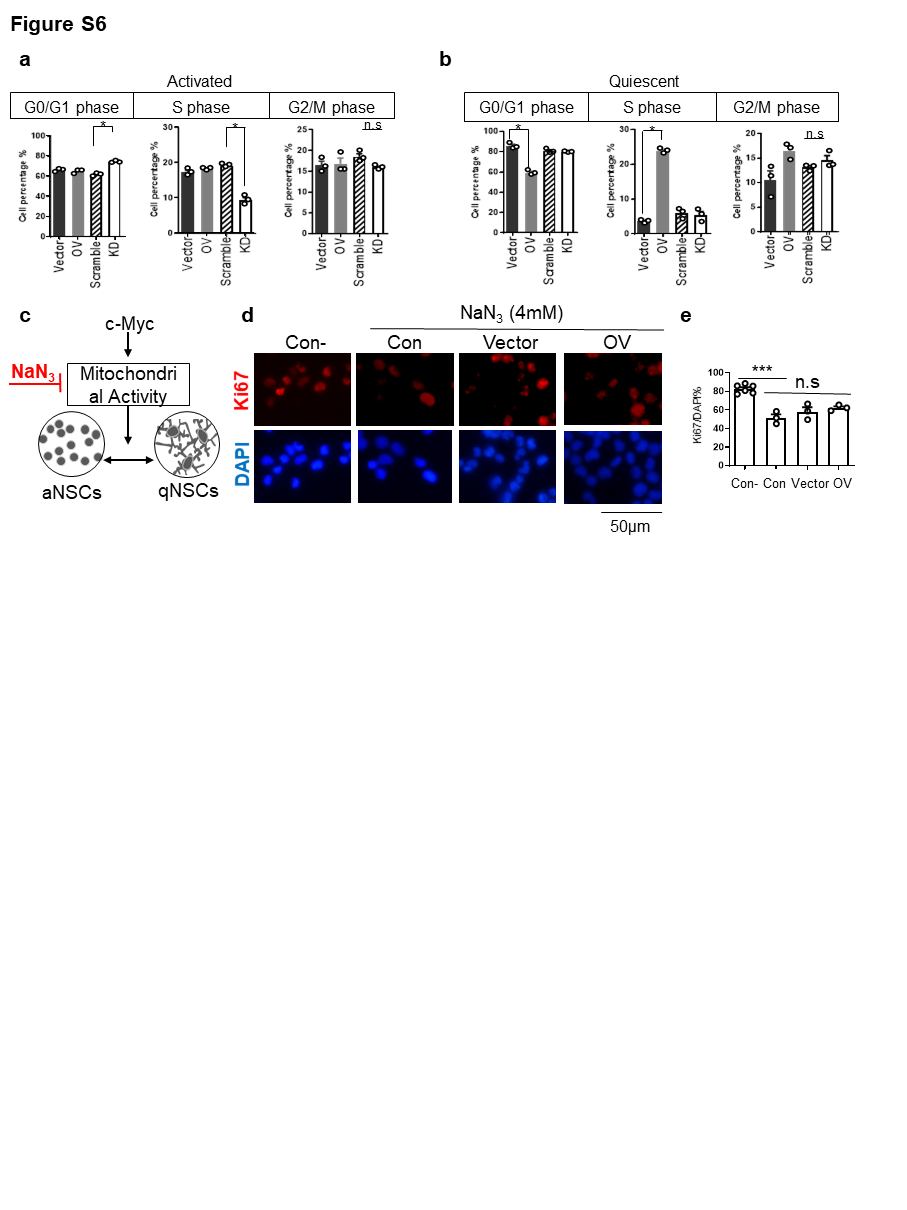

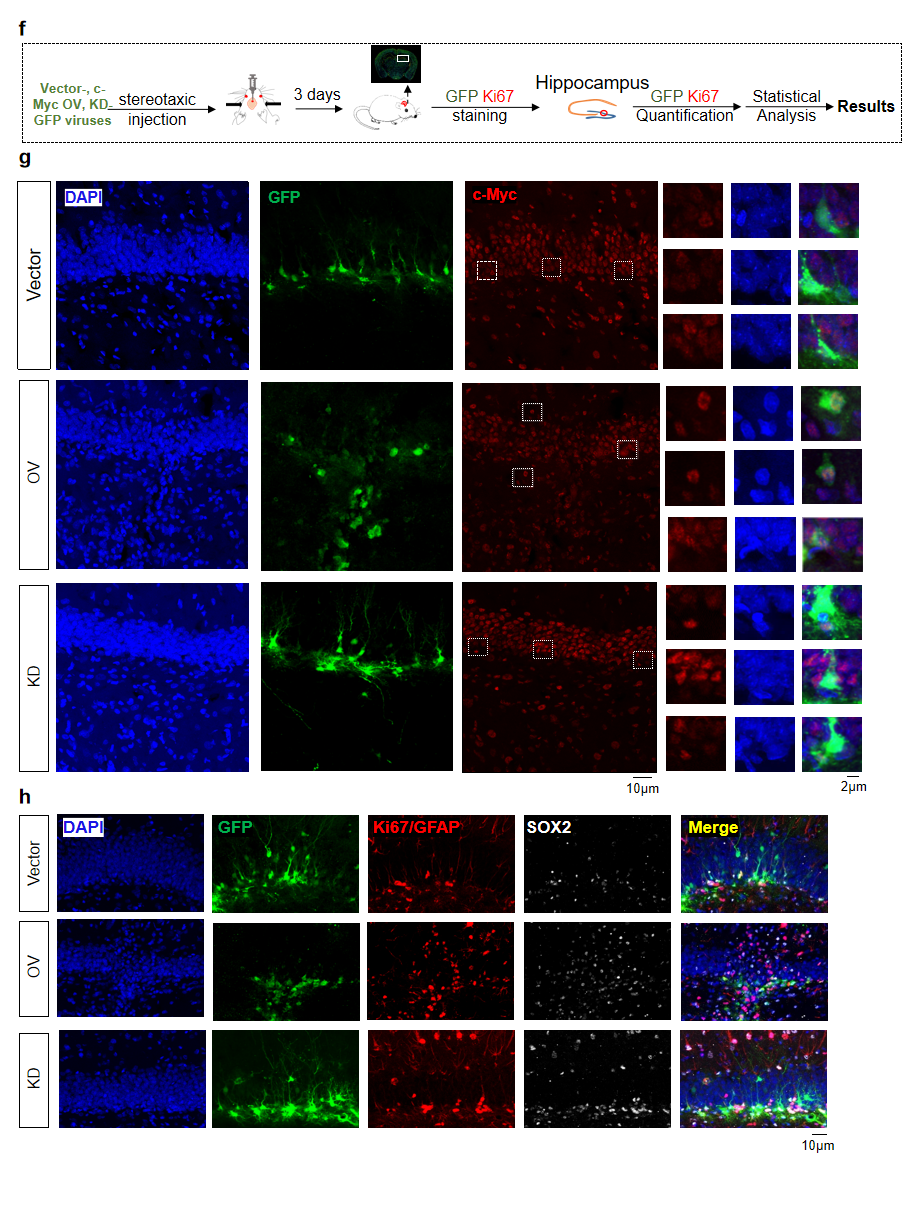
**

**Fig. S6 Control of NSC quiescence and proliferation *in vitro* by c-Myc. a** PY-Hoechst flow cytometric analysis of NSCs *in vitro* (n=3). An increase in G0-phase cells in c-Myc-KD aNSCs and a decrease in G0-phase cells in c-Myc-OV qNSCs were observed. **b** A decrease in S-phase population in c-Myc-KD aNSCs and an increase in S-phase population in c-Myc-OV qNSCs were observed. **c** Schematic of the mechanism by which NaN_3_ inhibits mitochondrial activity to block the function of c-Myc in NSCs. **d** Ki67 (red) and DAPI (blue) staining in NSCs with NaN_3_ treatment. **e** Ablation of the c-Myc-mediated increase in NSC proliferation by the mitochondrial activity inhibitor NaN_3_ (n=3). **f** Schematic of the procedure for determining the proliferation rate of the infected cells (GFP+) by co-staining GFP with Ki67 after stereotactic injections of vector, c-Myc OV- and KD-GFP viruses *in vivo*. **g** Confirmation of successful c-Myc overexpression and knockdown *in vivo* by co-staining c-Myc (red) with GFP (green). **h** Proliferation in viral infected cells (GFP+) detected by co-staining of GFP (green), Ki67 (red), GFAP (red), Sox2 (white) and DAPI (blue). All data are presented as the mean ± SEM values. An unpaired t-test was used to analyse the difference between the two groups. **P*<0.05; ***P*<0.01; ****P*<0.002; n.s, no significance.
